# Maternal nutritional status, decision-making autonomy and the nutritional status of adolescent girls: a cross-sectional analysis in the Mion District of Ghana

**DOI:** 10.1017/jns.2022.95

**Published:** 2022-11-02

**Authors:** Monicah Agaba, Fusta Azupogo, Inge D. Brouwer

**Affiliations:** 1 Wageningen University and Research, 239 Hoevestein, 6708AK Wageningen, The Netherlands; 2 Department of Family and Consumer Sciences, Faculty of Agriculture, Food and Consumer Sciences, University for Development Studies, Tamale, Ghana; 3 Division of Human Nutrition, Wageningen University and Research Centre, Stippeneng 4, 6708WE Wageningen, The Netherlands

**Keywords:** Adolescent girls, Adolescent nutritional status, Ghana, Height, Maternal decision-making, Maternal nutritional status

## Abstract

A mother's nutritional status and participation in household decision-making, a proxy for empowerment, are known determinants of improved nutrition and health outcomes for infants and young children; however, little is known about the association among adolescents. We examined the association between maternal nutritional status, decision-making autonomy and adolescent girls’ nutritional status. We analysed data of 711 mother–adolescent girl pairs aged 10–17 years from the Mion District, Ghana. Maternal nutritional status and decision-making autonomy were the independent variables while the outcomes were adolescent girls’ nutritional status as defined by anaemia, stunting and body mass index-for-age *Z*-score categories. Girl-level (age, menarche status and the frequency of animal-source food consumption), mother-level (age, education level, and monthly earnings) and household-level (wealth index, food security status and family size) covariates were adjusted for in the analysis. All associations were examined with hierarchical survey logistic regression. There was no association between maternal height and adolescent girls being anaemic, underweight or overweight/obese. Increasing maternal height reduced the odds of being stunted [adjusted odds ratio (OR) 0⋅92, 95 % CI (0⋅89, 0⋅95)] for the adolescent girl. Maternal overweight/obesity was positively associated with the girl being anaemic [OR 1⋅35, 95 % CI (1⋅06, 1⋅72)]. The adolescent girl was more than five times likely to be thin [OR 5⋅28, 95 % CI (1⋅64–17⋅04)] when the mother was underweight. Maternal decision-making autonomy was inversely associated with stunting [OR 0⋅88, 95 % CI (0⋅79, 0⋅99)] among the girls. Our findings suggest that intergenerational linkages of a mother's nutritional status are not limited to childhood but also during adolescence.

## Background

Undernutrition, overnutrition and rising evidence of metabolic disorders plague adolescents globally, all of which are linked to poor health outcomes in adulthood^(1)^. In the last 50 years, adolescents’ health has only improved marginally compared with younger children^(2)^, and Sub-Saharan Africa (SSA) is known to have the worst adolescent health profiles, with persistently high mortality from maternal and infectious causes^(3)^. In low- and middle-income countries (LMICs), there is currently a double burden of malnutrition for adolescents, with rising overweight/obesity and persistently high rates of undernutrition^(4,5)^.

When compared with their male counterparts, the double burden of malnutrition is significantly higher in females aged 5–19 years in SSA^(1)^. Varied and relatively high prevalence rates of stunting (2⋅1–50⋅3 %), underweight (7–19⋅4 %) and overweight/obesity (6⋅9–19⋅8 %) have been reported for Ghanaian adolescents depending on the context^(6–10)^. SSA is next to South Asia in the global burden of anaemia and iron deficiency anaemia for adolescent girls^(4)^. A recent survey from Ghana found about 24 % of adolescent girls aged 10–19 years anaemic^(11)^. Pre-pregnancy haemoglobin and iron status are key risk factors for anaemia-related morbidity and mortality during pregnancy^(12)^.

The deprivations girls encounter have intergenerational consequences for the nutrition and health of their future offspring^(13)^. Maternal height is associated with the attained height and general growth outcomes of a child^(14)^. Maternal height measures the accumulated nutritional status of a mother over the years, reflecting environmental and nutritional exposure during childhood and adolescence^(15)^. Short women have a small uterus which can cause the cervix to shorten and the surrounding membrane to enlarge, resulting in delivery problems, pre-term birth, low birth weight (<2⋅5 kg) babies and an increased risk of maternal mortality^(16)^. In addition, short maternal height raises the risk of morbidity and mortality in new-borns and young children^(17)^. However, data are scarce on whether these intergenerational impacts continue into adolescence. Nonetheless, Jaaskelainen and colleagues^(18)^ found that there is an intergenerational transmission of overweight/obesity between parents (father and mother) and children at the age of 16 years in a 16-year follow-up study in Finland; the association between parent and child in this study was about three times higher among girls than boys. One study in Brazil also found that mothers who were overweight had adolescent daughters who were either overweight or obese^(19)^.

Maternal participation in household decision-making, a proxy for empowerment, is a determinant of improved nutrition and health outcomes for infants and young children^(20,21)^. The literature reveals a strong link between women's empowerment and improvements in the nutrition and health of children under the age of five^(20,21)^. There is also an established association between women's empowerment and better dietary diversity in households^(22,23)^. In many rural areas in LMICs, women are in charge of making decisions regarding meal preparation and giving care to children under age five and other family members^(24)^. Women's empowerment in decision-making results in healthier eating habits and a more diverse diet^(25)^. According to the 2014 Ghana demographic health survey, 63 % of women who earn their own money can make independent decisions on how to spend it^(26)^. Until now, data on the association between maternal autonomy regarding participation in decision-making and the nutrition of children is generally limited to children under-five years of age^(20,21,23,24,27)^; emerging data on adolescents is largely from Bangladesh with mixed results^(14,28)^. In the present study, we analysed the association between a mother's nutritional status, and autonomy in household decision-making with the nutrition of adolescent girls in north-eastern Ghana using mother–daughter pairs.

## Method

### Study design and setting

We analysed baseline data from the Ten2Twenty-Ghana study; the study design, setting, and population have previously been described in detail elsewhere^(29)^. In brief, Ten2Twenty-Ghana was a randomised controlled trial evaluating the efficacy of multiple-micronutrient fortified biscuits compared with unfortified biscuits on micronutrient status, height and cognition of adolescent girls aged 10–17 years in the Mion District, in north-eastern Ghana. The study began with a large survey (*n* 1057), which led to a trial (*n* 621). The survey was conducted in November/December 2018; it includes data on the nutritional status of the girls, their time use, aspirations, and dietary intake, socio-economic status, household demographics, and structure, and maternal factors including nutritional status, participation in household decision-making and a life-history calendar that captured maternal fertility, education and occupation. Participation was entirely voluntary, and the girl gave her assent, after receiving signed/thumb-printed informed consent from her guardian or parent. The study protocol was approved by the Navrongo Health Research Centre Institutional Review Board (NHRCIRB323).

The research was carried out in the Mion District of Ghana's. The climate of the district is tropical, with two distinct seasons: a dry season from November to March and a rainy season from April to October. According to the 2010 Ghana population and housing census, Mion District has a population of 81 812, with 91⋅1 % of that population residing in rural areas; about 19⋅5 % of the district's female population is aged 10–19 years; the illiteracy rate is high in the district and the population is mostly dependent on agriculture as its livelihood^(30)^.

### Study population and population for analysis

**Fig. 1.**
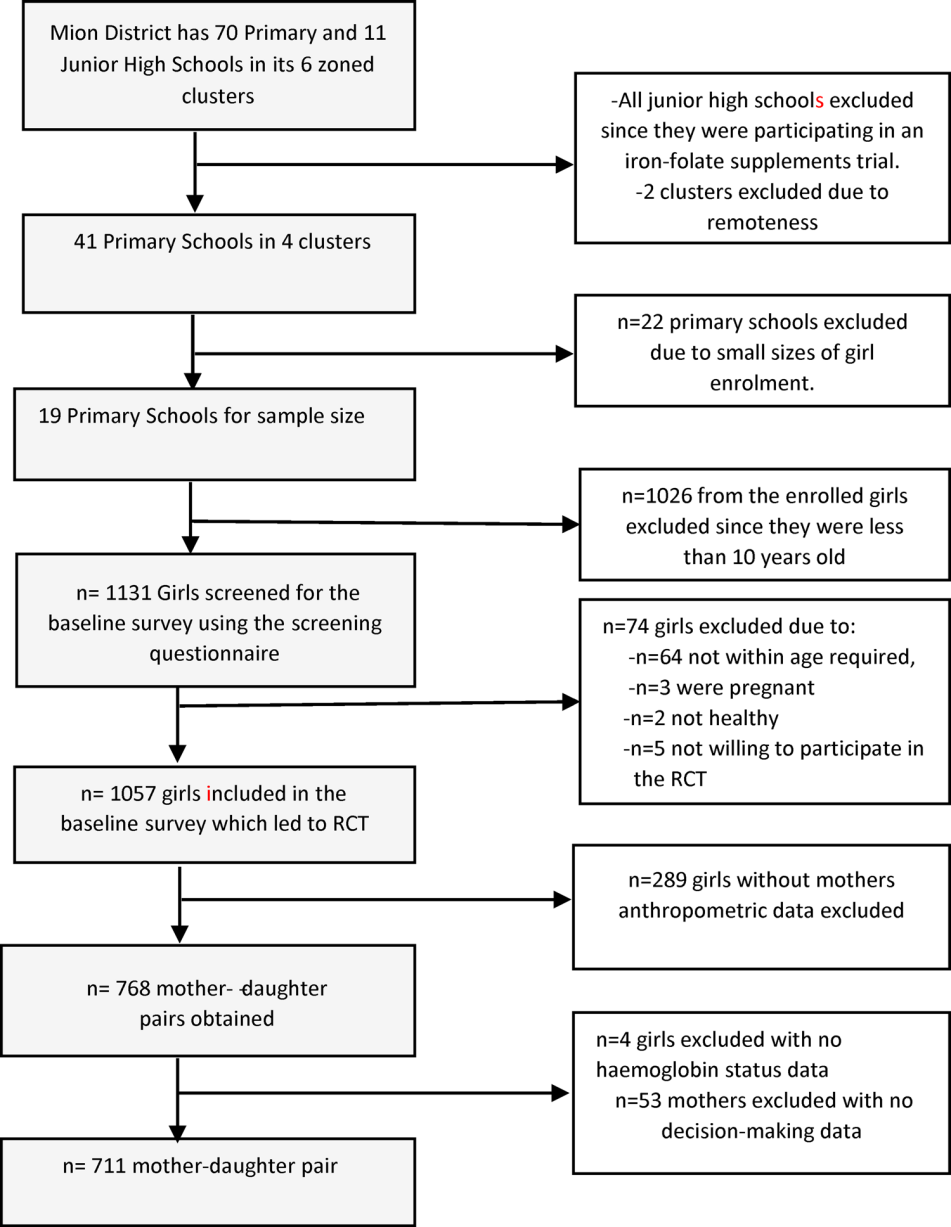
Flowchart showing the sample selection of the present study.

The study participants were adolescent girls aged 10–17 years and their mothers, residing in the Mion district, in Ghana's Northern region. The adolescent girls were selected from nineteen different elementary schools across the district. The sampling included four clusters, where four schools in the urban area were all selected, and fifteen larger rural schools were selected. A 16-item screening questionnaire ensured that all participating adolescent girls were pre- or post-menarche, healthy with no apparent signs of poor health, not pregnant and not lactating at the time of the survey^(29)^. Adolescent girls with missing data on haemoglobin (Hb) status (*n* 4) and mothers with missing data on decision-making (*n* 53) were excluded from the final analysis. Thus, a total of 711 mother–daughter pairs were used in the present study (Fig. 1).

### Data collection procedure

The data collection methods included one-on-one interviews with a semi-structured questionnaire, anthropometry, Hb status assessment by finger prick, a qualitative 24-hour dietary recall (24HR) and a one-month food frequency questionnaire (FFQ), conducted in November/December 2018. The questionnaire was pre-tested in the neighbouring Yendi Municipality. Given some questions like menarche were sensitive, interviewers were trained ladies, recruited from the University for Development Studies (UDS). Supervisors verified and validated all questionnaires for consistency and completeness throughout fieldwork.

### Independent variables

#### Mother's nutritional status

Standardised anthropometric guidelines^(31)^ were followed in measuring the height (cm) and weight (kg) of the mother in duplicates to the nearest 0⋅1 decimal using a Seca stadiometer and a digital weighing scale, respectively. The average of the duplicate measures was used in the analysis. Body mass index (BMI, kg/m^2^) of mothers was computed and BMI categories were defined: underweight (BMI < 18⋅5), normal weight (18⋅5 ≤ BMI < 25) and overweight/obese (BMI ≥ 25)^(31)^. The attained height of the mother was the mean height.

#### Maternal decision-making autonomy

The mothers’ participation in household decision-making autonomy, herein referred to as maternal autonomy, was assessed with the demographic and health survey 8-item final decision-making index^(32)^. These questions included final say on: (1) ‘how respondent's money is spent’, (2) ‘health care’, (3) ‘making large household purchases’, (4) ‘making household purchases for daily needs’, (5) ‘family visit’, (6) ‘food to be cooked each day’, (7) ‘what to do with money husband earns’ and (8) ‘the number of children to have’. We assigned a score of 1 to a mother involved in decision-making alone or with any other person in the household, whereas a score of 0 was given if she did not participate in decision-making. The scores from the 8-item questions were summed, ranging from 0 to 8, with a higher score denoting higher participation in decision-making in the household.

### Outcome variables

#### Anthropometry of the girl

The height (cm) and weight (kg) of the adolescent girl were also measured following the same guidelines described for the mother. We computed the height-for-age *Z*-score (HAZ) and body mass index-for-age *Z*-score (BAZ) of the girl with WHO AnthroPlus using the WHO growth reference for 10–19 years of age adolescent girls. We defined stunting as HAZ < −2sd, whereas BAZ was categorised as thinness (BAZ < −2sd), normal weight (−2sd ≤ BAZ ≤ + 1sd) and overweight/obese (BAZ > + 1sd)^(33)^. The attained height of the girl was the mean of the duplicate height measured.

#### Haemoglobin status of the girl

Phlebotomists from the Tamale Teaching Hospital assessed Hb by finger prick using a HemoCue 301 (Angelholm, Sweden; 0⋅1g/dl precision). The photometer was calibrated with certified quality control samples from the CDC/Atlanta, and the readings of ten patients were repeated each day for quality control. We defined anaemia as having Hb < 12 g/dl for girls aged ≥12 years and Hb < 11⋅5 for girls <12 years^(34)^.

### Covariates

#### Girl-level covariates

A single qualitative 24HR assessed the dietary diversity score (DDS) of the girls using a 10-food group indicator^(35)^. In the 24HR, the girl was first asked to mention all foods, including drinks and snacks that she consumed in and outside the home (including school) the previous day. She was then asked to describe the ingredients of any mixed dishes. Based on a pre-defined table with a list of all possible food items in the ten food groups, a score of 1, else 0 was given if a girl consumed at least one food item from any food group. A summated score was computed by summing the scores for all the food groups, resulting in a maximum attainable score of 10. The ten food groups included: grains, white roots, tubers and plantains (1), pulses (beans, peas and lentils) (2), nuts and seeds (3), dairy (4), meat, poultry and fish (5), eggs (6), dark green leafy vegetables (7), other vitamin A-rich fruits and vegetables (8), other vegetables (9) and other fruits (10). We next defined minimum dietary diversity (MDD-W) as DDS ≥5^(35)^. The effect of dietary diversity as a continuous score (DDS) and as a dichotomous variable (MDD-W) was also explored. Additionally, the girls’ dietary patterns were assessed with a 1-month FFQ using the ten food groups^(35)^. The data also included the ethnicity, religion, class and age of the girl using a household roster and as well, menarche status based on recall.

#### Maternal-level covariates

Maternal covariates in the data included education (none, basic, secondary/higher), occupation (not currently working, farmer, trader and others), literacy (dichotomous) and age. A life-history calendar also tracked the mother's parity and earnings in a month.

#### Household-level covariates

The international wealth index (IWI) was used to assess the socio-economic status of the households^(36)^. The IWI ranks households based on the ownership of durable assets including TV, refrigerator, phone, bicycle, car, household utensils categorised as cheap (<$40) and expensive (>$250), access to electricity, the type of water and toilet facilities accessed by the household and as well as the floor material of the household. The IWI was created purposely for assessing the socio-economic status of households in LMICs using principal component analysis (PCA) on data from 97 LMICs^(36)^. We adopted and used the IWI SPSS Syntax to run the calculations; the IWI ranges from a minimum of 25 to a maximum of 100. Households were subsequently ranked into quintiles of wealth based on their IWI score.

The Food Insecurity Experience Scale (FIES)^(37)^ was used to measure the food security of the girls’ households. The FIES is an 8-question survey that uses yes/no responses to assess the degree of food insecurity. When the answer is ‘yes’, the questions are given a score of 1; otherwise, they are given a score of 0. We computed the FIES score by summing the scores of the eight items; the score ranged between 0 and 8. A higher score indicated a more severe level of food insecurity, whereas a lower score indicated a less severe level of food insecurity. The sum score was used to assign the girls to one of the following categories: food secure (FIES = 0), mild food insecure (FIES score 1–3), moderate food insecure (FIES score 4–6) and severe food insecure (FIES score 7–8). A household roster captured data on paternal education (none, primary, secondary/higher), occupation (none, farmer, trader/self-employed, formal employee) and literacy (dichotomous). We computed and included in our analyses household dependency ratio, sex and literacy ratios similar to the Ghana Statistical Service^(30)^. Count variables for household size and the number of children under 5 years were also explored.

### Statistical analysis

The statistical software programs SPSS (version 26) and R-studio (version 4.0.0) were used to analyse the data. Categorical descriptive variables were expressed as percentages and frequencies, while continuous variables were presented as means and standard deviation (mean ± sd). Data normality was examined visually with the normality histogram curves and Q-Q plots.

We assessed the association between maternal nutritional status, autonomy and the nutrition of the adolescent girls using survey logistic regression (binary and multinomial); including a random intercept for the study design (School). The outcome variables in the binary logistic regression analysis were anaemia (anaemic or not) and stunting status (stunted or not), while the BAZ category (normal, thin, overweight/obese) of the girl was the outcome variable for the multinomial logistic regression. The mother's attained height, BMI category (normal, underweight and overweight/obese) and autonomy (as a continuous variable) were the exposure variables. We categorised the height of the mother into a dichotomous variable as short stature (<145 cm height) and normal stature (≥145)^(31)^ but short stature prevalence (0⋅6 %) was low; hence, we analysed height (cm) only as a continuous variable. In the analysis, maternal height and autonomy were analysed together and the BMI category of the mother replaced height in a repeated analysis. The crude and adjusted odds ratios and 95 % confidence intervals with their corresponding *P*-values were presented. A two-tailed *P*-value ≤ 0⋅05 at a 95 % confidence interval was considered statistically significant. Potential confounding variables were selected *a priori* based on literature and included girl-level (age, menarche status, dietary diversity and/or animal-sourced food intake), maternal (age, education, monthly earnings) and household (food security, wealth index, household size) factors^(14,17,18,20–25)^. Multicollinearity between explanatory variables was assessed using tolerance values (TOL) < 0⋅1 and the variance inflation factor (VIF) < 10 in a linear regression step. Aside from the basic model (model 1), three multivariable models were developed. Model 2 was adjusted for adolescent girl-level characteristics such as the girl's age, menarche status, DDS and frequency of animal-source food intake in a hierarchical order. Model 3 took into account other maternal factors such as age, education and monthly wages. Finally, household-level factors such as household food security, wealth index and family size were adjusted for in model 4. We looked for pair-wise interaction terms between maternal decision-making and the adolescent girl's other explanatory variables, such as DDS and animal-sourced food intake, but none was found to be significant. Mathematically, the models are expressed below.

Model 1 (Crude model): 

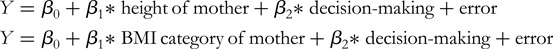


Model 2: adjusted for potential child-level covariates.
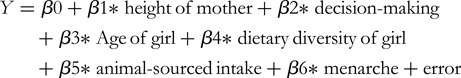

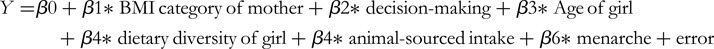


Model 3: adjusted for potential maternal covariates.
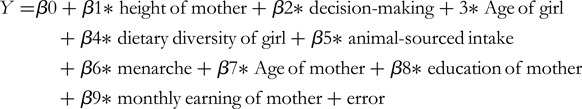

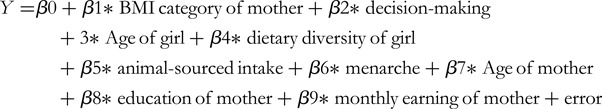


Model 4: adjusted for potential household-level covariates.
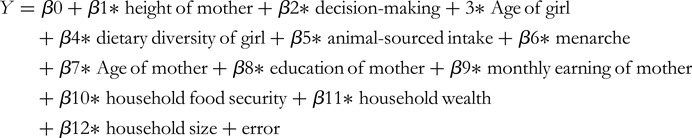

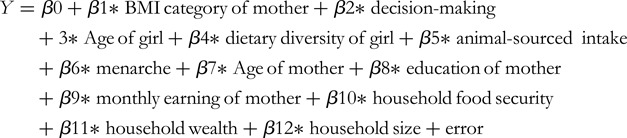


### Sensitivity analysis

We repeated all the analyses using linear mixed model analysis, including a random intercept of the study design (school) in which the Hb status (g/dl), HAZ and the BAZ of the girl were the outcomes (Supplementary Tables S1 and S2).

## Results

### Socio-demographic characteristics of the study population

Table 1 shows the descriptive statistics of the 711 adolescent girls. The average age of adolescent girls was 12⋅5 ± 1⋅9 years, whereas that of their mothers was 39 ± 7⋅4 years. About 81 % of the girls were pre-menarche at the time of screening. Table 1 also demonstrates that the majority of mothers were farmers (75 %) and non-literate (95 %). A majority (59 %) of the girls were of Dogomba ethnic origin, and the majority (60 %) were Muslims. Only one-fifth of the homes were categorised as food secure with approximately 20 % having severe food insecurity and about 19 % having a high socio-economic position. Lastly, the average household size was 11⋅9 ± 5⋅0 people.

**Table 1.** Socio-demographic characteristics of the adolescent girls

Variable	Mean or frequency (Percentage)	sd
Girl-level characteristics		
Age of adolescent girls (mean, sd)	12⋅5	1⋅9
Girl is post-menarche (%)	114 (18⋅8)	
Ethnic group (%)		
Dogomba	361 (58⋅8)	
Konkomba	247 (40⋅2)	
Others	6 (1⋅0)	
Religion^a^ (%)		
Muslim	370 (60⋅4)	
Christian	169 (27⋅6)	
Others	74 (12⋅0)	
Maternal characteristics		
Age of mothers (mean, sd)	39	7⋅4
Maternal occupation^b^ (%)		
Not currently working	25 (3⋅9)	
Farmer	483 (74⋅8)	
Trader	116 (18⋅0)	
Others	22 (3⋅4)	
Level of education of mother^c^ (%)		
None	10 (1⋅6)	
Basic	605 (94⋅1)	
Secondary/higher	28 (4⋅4)	
Mother is literate (%)	617 (95⋅7)	
Parity (mean, sd)	6⋅6	2⋅4
Maternal earnings (%)		
Low (<GHc100)	204(37)	
Moderate(GHc100–200)	134(24)	
High (>GHc200)	210(38)	
Household characteristics		
Paternal occupation^b^ (%)		
Not currently working	25 (4⋅1)	
Farmer	531 (87⋅8)	
Trader	32 (5⋅3)	
Others	17 (2⋅8)	
Level of education of father^c^ (%)		
None	35 (6⋅0)	
Primary	531 (90⋅5)	
Secondary/Higher	21 (3⋅6)	
Socio-economic status (SES) (%)		
Very low SES	136 (20⋅4)	
Low SES	119 (17⋅8)	
Medium SES	109 (16⋅3)	
High SES	176 (26⋅3)	
Very high SES	128 (19⋅2)	
Household food insecurity (%)		
Food secure	135 (19⋅1)	
Mild food insecurity	182 (25⋅7)	
Moderate food insecurity	251 (35⋅5)	
Severe food insecurity	139 (19⋅7)	
Household size (mean, sd)	11⋅9	5⋅0

a Under the variable religion, African Traditional; religion (ATR), and other religions were combined to form others.

b Under occupation, we merged the self-employed, salary worker into others.

c Under education level attained, Kindergarten and Primary levels were combined into Basic, and Middle/JSS/JHS, SSS/SHS and Tertiary were merged as secondary level/higher.

### Adolescent nutritional status and dietary intake

The average HAZ and BAZ of the girls were −0⋅9 ± 1⋅2 and −0⋅7 ± 0⋅9, respectively, as shown in Table 2. Out of the 711 adolescents, 19⋅0 % were classified as stunted, 9⋅0 % as thin and 2⋅7 % as overweight/obese. The girls’ average Hb level was 12⋅0 ± 1⋅2 g/dl, with roughly 39 % of them anaemic. Table 2 further reveals that the mean DDS of the girls was 6⋅0 ± 1⋅2 out of ten food groups. Based on the FFQ, the girls ate animal-sourced foods on an average of 8⋅9 ± 4⋅0 d in the previous month. The average consumption of iron-rich foods, vitamin A-rich foods, and fruits and vegetables in the last month was 15⋅7 ± 3⋅4, 11⋅0 ± 3⋅3 and 12⋅3 ± 6⋅2 d, respectively. Table 2 also shows that the most frequently consumed foods were cereals and grains, consumed almost daily. The fish food group was the second most frequently consumed food group (23⋅4 ± 9⋅6 d), mainly attributed to the consumption of anchovies. Eggs and sugars were the least frequently consumed foods.

**Table 2.** Nutritional status and dietary characteristics of the girls

Variable	Mean	sd
Height-for-age *Z*-score	−0⋅9	1⋅2
Stunted (%)^a^	137 (19⋅3)	
Body mass index-for-age *Z*-score (BAZ)	−0⋅7	0⋅9
BAZ category of the girl (%)^a^		
Thinness	64 (9⋅0)	
Overweight/obese	19 (2⋅7)	
Haemoglobin status (g/dl)	12⋅0	1⋅2
Anaemic (%)^a^^,^b^^	276 (38⋅8)	
Dietary diversity	6⋅0	1⋅2
Mean frequency of food group consumption in the past month		
Animal-source foods	8⋅9	4⋅0
Iron-rich foods	15⋅7	3⋅4
Vitamin A-rich foods	11⋅0	3⋅3
Fruits and vegetables	12⋅3	6⋅2
Fruit	6⋅3	6⋅0
Vegetables	18⋅2	10⋅5
Sweets	9⋅2	8⋅3
Sugars	2⋅7	5⋅1
Savoury/snacks	10⋅6	8⋅1
Dairy	4⋅2	6⋅4
Meat	5⋅8	6⋅9
Eggs	2⋅4	4⋅6
Fish	23⋅4	9⋅6
Pulses/nuts	17⋅7	8⋅3
Cereals/grains	29⋅6	2⋅9

Unless otherwise stated, values are means, sd.

a Values are frequency and percentages in the brackets.

^b^ Hb <12⋅0 g/dl for girls aged > 12 years and Hb < 11⋅5g/dl for girls aged < 12 years.

### Maternal nutritional status and decision-making autonomy

The mothers had a mean height of 158 ± 5⋅6 cm, with 0⋅6 % having short stature (<145 cm). Furthermore, the mothers’ average BMI was 22⋅8 ± 3⋅7 kg/m^2^. About 6⋅4 % of the mothers had chronic energy deficiency (BMI < 18⋅5 kg/m^2^), 17 % were overweight (>25 kg/m^2^) and 5 % were obese. Overall, the mean decision-making autonomy score of the mothers was 5⋅4 ± 1⋅4, out of a possible total of 8 (Table 3). When compared with mothers from food secure households, the prevalence of chronic energy deficiency was about twice among mothers from food insecure households (7⋅3 % *v*. 3⋅0 %, *P* = 0⋅06).

**Table 3.** The nutritional status and decision-making autonomy of the mothers

Variable	Frequency (Percentage)	sd
BMI of the mother (mean, sd, kg/m^2^)	22⋅8	3⋅7
BMI category of the mother (%)		
Underweight	45 (6⋅4)	
Normal	502 (71⋅6)	
Overweight	117 (16⋅7)	
Obese	37 (5⋅3)	
Height of mother (mean, sd, cm)	158⋅8	5⋅6
Short stature (%)	4 (0⋅6)	
Maternal decision-making	5⋅4	1⋅4

Unless otherwise stated, values are frequencies and percentages in brackets.

### Association between maternal height and the nutritional status of the adolescent girls

We found no association between maternal height and adolescent girls being anaemic, thin or overweight; controlling for possible confounding variables did not affect the results (Table 4). However, in the crude model, a unit increase in maternal height was associated with 8 % reduced odds of being stunted [OR = 0⋅92, 95 % CI (0⋅89, 0⋅95)] among the girls; adjustment for probable confounding factors did not affect the observed association (Table 4).

**Table 4.** Logistic regression analysis of the association between maternal height, decision-making autonomy and adolescent girls’ nutritional status

Statistical model	Anaemia		Stunting		Thinness		Overweight	
	OR (95 % CI)	*P*-value	OR (95 % CI)	*P*-value	OR (95 % CI)	*P*-value	OR (95 % CI)	*P*-value
Crude model: Model 1								
Maternal height (cm)	0⋅99(0⋅96–1⋅02)	0⋅65	0⋅92(0⋅89–0⋅95)	<0⋅0001	0⋅97(0⋅92–1⋅01)	0⋅16	1⋅02(0⋅94–1⋅10)	0⋅63
Decision-making	0⋅92(0⋅82–1⋅04)	0⋅17	0⋅88(0⋅79–0⋅99)	0⋅03	0⋅89(0⋅75–1⋅07)	0⋅24	0⋅99(0⋅71–1⋅37)	0⋅93
Model 2								
Maternal height (cm)	0⋅99(0⋅96–1⋅02)	0⋅57	0⋅92(0⋅90–0⋅95)	<0⋅0001	0⋅97(0⋅92–1⋅02)	0⋅36	1⋅02(0⋅98–1⋅05)	0⋅21
Decision-making	0⋅98(0⋅87–1⋅11)	0⋅73	0⋅84(0⋅74–0⋅95)	0⋅01	0⋅90(0⋅73–1⋅12)	0⋅35	0⋅98(0⋅69–1⋅40)	0⋅92
Model 3								
Maternal height (cm)	0⋅99(0⋅96–1⋅02)	0⋅62	0⋅92(0⋅88–0⋅97)	0⋅01	0⋅98(0⋅91–1⋅04)	0⋅44	0⋅98(0⋅86–1⋅12)	0⋅78
Decision-making	0⋅91(0⋅77–1⋅07)	0⋅21	0⋅89(0⋅72–1⋅11)	0⋅25	0⋅85(0⋅66–1⋅09)	0⋅21	0⋅95(0⋅58–1⋅55)	0⋅83
Model 4								
Maternal height (cm)	0⋅99(0⋅96–1⋅02)	0⋅35	0⋅91(0⋅89–0⋅95)	0⋅0002	0⋅97(0⋅90–1⋅03)	0⋅32	1⋅01(0⋅87–1⋅19)	0⋅88
Decision-making	0⋅89(0⋅82–1⋅08)	0⋅32	0⋅90(0⋅79–1⋅00)	0⋅05	0⋅82(0⋅63–1⋅06)	0⋅14	0⋅93(0⋅54–1⋅60)	0⋅80

Anaemia and stunting were analysed using logistic regression, whereas the BAZ category (thinness, normal weight and overweight) was analysed using multinomial logistic regression. For BAZ categories, the normal was used as the reference category. Model 1 was the crude model; Model 2 was adjusted for adolescent-level covariates including age, menarche, dietary diversity and frequency of animal-source intake; Model 3 was a further adjusted model for maternal-level covariates including the age of the mother, their monthly earnings and education. Model 4 was finally adjusted for household-level covariates, including wealth index, food insecurity and household size.

### Association between the nutritional status of the mother and the nutritional status of the adolescent girls

The findings in Table 5 showed that overweight/obesity of the mother significantly increased the odds of being anaemic among the girls by 35 % and the observed association remained in the final adjusted model [OR 1⋅55, 95 % CI (1⋅06, 1⋅93)]. However, overweight/obesity of the mother increased the odds of being overweight/obese among the girls only slightly. Likewise, there was no association between maternal overweight/obesity and the odds of being stunted or thin among the girls in both the crude and adjusted models.

**Table 5.** Logistic regression analysis of the association between maternal body mass index category, decision-making autonomy and adolescent girls’ nutritional status

Statistical model	Anaemia		Stunting		Thinness		Overweight	
	OR (95 % CI)	*P*-value	OR (95 % CI)	*P*-value	OR (95 % CI)	*P*-value	OR (95 % CI)	*P*-value
Crude model: Model 1								
Maternal BMI								
Normal (ref)	Ref		Ref		Ref		Ref	
Underweight	0⋅99(0⋅51–1⋅93)	0⋅98	0⋅89(0⋅35–2⋅20)	0⋅78	3⋅18(1⋅50–6⋅73)	0⋅002	1⋅12(0⋅14–8⋅89)	0⋅91
Overweight/obese	1⋅35(1⋅06–1⋅72)	0⋅02	0⋅98(0⋅61–1⋅59)	0⋅95	0⋅47(0⋅21–1⋅06)	0⋅07	1⋅57(0⋅58–4⋅27)	0⋅37
Decision-making	0⋅93 (0⋅82–1⋅06)	0⋅28	0⋅89(0⋅78–1⋅00)	0⋅05	0⋅89(0⋅75–1⋅07)	0⋅22	0⋅99(0⋅71–1⋅37)	0⋅95
Model 2								
Maternal BMI								
Normal (ref)	Ref		Ref		Ref		Ref	
Underweight	1⋅00(0⋅45–2⋅20)	0⋅95	0⋅94(0⋅34–2⋅59)	0⋅89	3⋅53(1⋅51–8⋅26)	0⋅003	1⋅00(0⋅96–1⋅97)	0⋅91
Overweight/obese	1⋅39(1⋅03–1⋅88)	0⋅03	1⋅04(0⋅63–1⋅70)	0⋅86	0⋅57(0⋅23–1⋅42)	0⋅23	1⋅69(0⋅55–5⋅24)	0⋅36
Decision-making	0⋅99(0⋅87–1⋅13)	0⋅86	0⋅84(0⋅74–0⋅95)	0⋅01	0⋅90(0⋅73–1⋅12)	0⋅34	0⋅99(0⋅69–1⋅41)	0⋅96
Model 3								
Maternal BMI								
Normal (ref)	Ref		Ref		Ref		Ref	
Underweight	1⋅22(0⋅55–2⋅70)	0⋅57	0⋅67(0⋅17–2⋅65)	0⋅49	4⋅79(1⋅71–13⋅41)	0⋅004	1⋅00(0⋅17–1⋅70)	0⋅77
Overweight/obese	1⋅34(1⋅07–1⋅85)	0⋅02	1⋅09(0⋅49–2⋅39)	0⋅78	0⋅65(0⋅21–2⋅05)	0⋅13	2⋅76(0⋅53–1⋅42)	0⋅11
Decision-making	0⋅91(0⋅77–1⋅06)	0⋅18	0⋅91(0⋅73–1⋅13)	0⋅29	0⋅82(0⋅64–1⋅06)	0⋅07	1⋅03(0⋅61–1⋅70)	0⋅75
Model 4								
Maternal BMI								
Normal (ref)	Ref		Ref		Ref		Ref	
Underweight	1⋅14(0⋅37–2⋅04)	0⋅71	0⋅63(0⋅31–2⋅38)	0⋅73	5⋅28(1⋅64–17⋅04)	0⋅005	1⋅00(0⋅10–10⋅13)	0⋅78
Overweight/obese	1⋅55(1⋅06–1⋅93)	0⋅03	1⋅29(0⋅55–1⋅67)	0⋅86	0⋅64(0⋅18–2⋅26)	0⋅49	2⋅22(0⋅25–1⋅95)	0⋅47
Decision-making	0⋅90(0⋅82–1⋅10)	0⋅46	0⋅91(0⋅78–1⋅01)	0⋅07	0⋅77(0⋅58–1⋅01)	0⋅06	0⋅86(0⋅43–1⋅72)	0⋅66

Anaemia and stunting were analysed using logistic regression whereas, the BAZ category (thinness, normal weight and overweight) was analysed using multinomial logistic regression. For BAZ categories, the normal was used as the reference category. Model 1 was the crude model; Model 2 was adjusted for adolescent-level covariates including age, menarche, dietary diversity and frequency of animal-source intake; Model 3 was a further adjusted model for maternal-level covariates including the age of the mother, their monthly earnings and education. Model 4 was finally adjusted for household-level covariates, including wealth index, food insecurity and household size.

Furthermore, in both the crude and adjusted models, the adolescent girl was more than five times more likely to be thin when her mother was underweight (Table 5). However, we found no association between a mother being underweight and the odds of anaemia, stunting and overweight/obesity among the adolescent girls.

### Association between maternal decision-making autonomy and the nutritional status of the adolescent girls

In our study, maternal decision-making autonomy was not associated with anaemia, thinness and overweight among the adolescent girls even after adjusting for possible confounding variables. However, the decision-making autonomy of the mother significantly reduced the odds of stunting among the adolescent girls by 12 % [OR 0⋅88, 95 % CI (0⋅79, 0⋅99)]; but the association was slightly attenuated in the final model [OR 0⋅90, 95 % CI (0⋅79, 1⋅00)] (Table 4). When replacing maternal height with the BMI category, the association between maternal decision-making autonomy and stunting was marginal in the crude [OR 0⋅89, 95 % CI (0⋅78, 1⋅00)] and final adjusted model [OR 0⋅91, 95 % CI (0⋅78, 1⋅01)]. A marginal association for reduced odds of thinness was also observed for decision-making autonomy in the final model [OR 0⋅77, 95 % CI (0⋅58, 1⋅01)] (Table 5).

### Sensitivity analysis using the linear mixed-effect model

In our sensitivity analysis with linear mixed methods (Supplementary Table S1 and S2), a unit increase in maternal height was positively associated with the HAZ of the girl (*β* = 0⋅04 ± 0⋅01, *P* < 0⋅0001) but the observed association was attenuated after controlling for probable confounding variables. Maternal overweight/obesity significantly increased the HAZ (*β* = 0⋅20 ± 0⋅05, *P* = 0⋅0004) and BAZ (*β* = 0⋅16 ± 0⋅04, *P* = 0⋅0002) of the girl in the crude model but the observed associations did not remain after adjusting for possible confounders. A unit increase in maternal decision-making autonomy significantly decreased the Hb status, HAZ and BAZ of the girl in the crude models but none of these associations remained statistically significant after adjustment for confounding variables.

## Discussion

The present study assessed the association between maternal nutritional status, decision-making autonomy and the nutrition of adolescent girls using survey data from the Ten2Twenty-Ghana study. Our findings suggest that intergenerational linkages of the mother's nutritional status are not limited to childhood but also during adolescence. Overall, our findings suggest that a higher attained maternal height reduces stunting among adolescent girls. Surprisingly, maternal overweight/obesity was positively associated with the adolescent girl being anaemic, and maternal underweight was associated with thinness among the adolescent girls. Our findings also support the school of thought that the ability of the mother to make decisions minimises stunting in her children.

Similar to our finding, Benny *et al.*^(38)^ reported that there was an increased risk of stunting among adolescents whose mothers had short stature. A pooled analysis conducted in five birth cohorts also showed that short mothers were more likely to have children who were short for their age at 2 years and shorter as adults^(39)^. Maternal height is protective against stunting as it is argued that mothers with normal height have better nutrition and health outcomes than mothers with short stature^(39)^. However, we found no evidence of an association between maternal height and being anaemic, thin or overweight among adolescent girls. A comparison of this finding is difficult due to the scarcity of data on the intergenerational effects of maternal nutrition on adolescents. However, a study in India among children below 5 years showed a marginal and small decrease in the risk of anaemia with a unit increase in maternal height^(17)^; this was partly attributed to the poor living conditions of mothers with short stature.

The prevalence of overweight/obesity (2⋅7 % *v*. 11⋅8 %) in the present study was lower compared with the national average for girls but thinness (9⋅0 % *v*. 1⋅6 %) was higher^(40)^. Overall, dietary patterns in Ghana are shifting, with noticeable differences between urban and rural settings^(41)^. Given that over 90 % of the Mion district is rural, it partly explains the findings on the relatively low overweight/obesity prevalence as urban areas are known to have a higher prevalence of overweight and obesity^(42,43)^. According to the literature, if a mother is overweight or obese, her teen daughter is likely to be as well^(19,44–46)^. The low prevalence of overweight/obesity among the girls may explain why maternal overweight/obesity was not associated with overweight/obesity among the adolescent girls in our study. However, in our study, the adolescent girl was more than thrice likely to be thin when the mother was underweight. Similar to our results, maternal underweight has been reported as a determinant of thinness among adolescents in Bangladesh^(28)^. Although the mechanism underlying maternal underweight and adolescent thinness is unknown, it might be argued that socio-cultural and economic factors are affiliated with the current findings since undernutrition is often associated with poor socio-economic conditions^(28,43)^. Furthermore, restricted household finances may reduce the family's purchasing power for a nutrient-rich diet, resulting in household food insecurity and raising the adolescent girl's likelihood of being thin. The lack of association between maternal overweight/obesity and that of the adolescent girl may also be that other households and environmental factors are accountable for overweight and obesity among the girls. Overweight and obesity, for example, have been linked to increased intake of fat-laden ‘fast foods’, rising consumption of sugar-sweetened carbonated drinks and sedentary lifestyles^(9,47)^.

The positive association between maternal overweight/obesity and anaemia among the adolescent girls in our study is contrary to the general understanding because anaemia is a micronutrient deficiency that often occurs in the context of undernutrition^(48)^. For example, anaemia is more common in developing contexts and it is often associated with low socio-economic status^(43)^. There are currently no studies on the link between maternal overweight/obesity and micronutrient deficiencies in teenagers, and the mechanism underlying the link is unknown. However, it is thought that maternal obesity during pregnancy negatively affects the newborn iron status via inflammatory pathways^(49)^. Given the rising prevalence of overweight and obesity among reproductive-age women in Ghana^(50)^, intervention programmes aimed at preventing anaemia in children and adolescents may also include measures to avoid overweight and obesity among mothers.

In contrast to Dieffenbach *et al.*^(51)^, the present study found no evidence of a link between the mother's BMI category and the adolescent girl's stunting. It is possible that, unlike attained height, the mother's BMI category does not adequately explain her long-term nutritional exposure, and, therefore, does not explain the adolescent's long-term nutritional status.

Our findings suggest that women's empowerment in terms of decision-making autonomy may have long-term favourable implications on adolescent nutrition. According to the literature, women's empowerment is associated with adolescent nutritional status^(28)^, and a mother's ability to make decisions is associated with the height attained by a girl^(52)^. In the present study, the decision-making autonomy of the mother as a proxy of women's empowerment was associated with decreased odds of being stunted among adolescent girls. It is argued that women's involvement in decision-making at the household level is essential for consuming a diverse diet and improving their children's nutrition outcomes^(22,23,25)^, which now links to adolescents. Nutrition-sensitive interventions should harness male involvement in empowerment programmes, sharing the advantages of involving their women in household decision-making.

Some limitations in the present study should be considered when interpreting our findings. The study's cross-sectional design does not allow for causal inferences between maternal nutritional status, decision-making and adolescent nutritional status; a prospective design would better address this. We, therefore, limit our findings to the description of observed associations. The empowerment index used only the decision-making index of the mother, which is not fully representative of the empowerment concept, reducing the predictive value of empowerment to decision-making participation. Nevertheless, several studies have shown that the decision-making of a mother improves the dietary intake and nutrition outcomes of children^(20,21,24,27)^. The study's findings might not be extrapolated to the whole of Ghana or to include boys as only girls were sampled. Although the present study included only school-going adolescent girls, girl-child school enrolment in Ghana has been over 85 % since 2013^(53)^. Accordingly, the study population may, therefore, represent all rural adolescent girls in northern Ghana and similar settings.

## Conclusion

Our findings suggest that improvements in the nutrition of a mother may have some positive effects on the nutrition of adolescents. Our findings also suggest that maternal overweight and obesity may contribute to anaemia among adolescents. As well, a mother's participation in household decision-making may improve nutrition and reduce stunting among adolescent girls.

## Supplementary material

For supplementary material accompanying this paper visit https://doi.org/10.1017/jns.2022.95. click here to view supplementary material
