# Supplementary material for: Maternal nutritional status, decision-making autonomy and the nutritional status of adolescent girls: a cross-sectional analysis in the Mion District of Ghana
Source: J Nutr Sci. 2022 Nov 2;11:e97. doi: 10.1017/jns.2022.95 (PMC9641508; doi:10.1017/jns.2022.95)
Supplement: Supplementary file 1 [file S2048679022000957sup001.docx]

## **Table S1: Sensitivity analysis showing the association between maternal height, decision-making autonomy, and adolescent nutritional status**

| **Statistical model** | **Hb status** | | **HAZ** | | **BAZ** | |
| --- | --- | --- | --- | --- | --- | --- |
|  | **β ± S.E** | **P-value** | **β ± S.E** | **P-value** | **β ± S.E** | **P-value** |
| **Model 1: crude** |  |  |  |  |  |  |
| Maternal height (cm) | -0.004 ± 0.008 | 0.58 | 0.04 ± 0.01 | <0.0001 | 0.003 ± 0.01 | 0.56 |
| Decision-making | 0.04 ± 0.03 | 0.20 | 0.04 ± 0.03 | 0.26 | 0.02 ± 0.02 | 0.48 |
| **Model 2** |  |  |  |  |  |  |
| Maternal Height (cm) | 0.00 ± 0.00 | 0.55 | 0.00 ± 0.00 | 0.45 | 0.00 ± 0.00 | 0.54 |
| Decision-making | 0.00 ±0.002 | 0.17 | 0.00 ± 0.002 | 0.16 | 0.00 ± 0.00 | 0.15 |
| **Model 3** |  |  |  |  |  |  |
| Maternal Height (cm) | 0.004 ± 0.004 | 0.46 | 0.004 ± 0.005 | 0.46 | 0.004 ± 0.005 | 0.38 |
| Decision-making | -0.05 ± 0.03 | 0.11 | -0.03 ± 0.03 | 0.44 | -0.03 ± 0.03 | 0.37 |
| **Model 4** |  |  |  |  |  |  |
| Maternal Height (cm) | 0.00 ± 0.00 | 0.97 | 0.00 ± 0.001 | 0.78 | 0.00 ± 0.001 | 0.96 |
| Decision-making | 0.01 ± 0.01 | 0.24 | 0.01 ± 0.01 | 0.16 | 0.01 ± 0.01 | 0.20 |

Model 1 was the crude model; Model 2 was adjusted for adolescent level covariates including age, menarche, dietary diversity, and frequency of animal source intake; Model 3 was a further adjusted model for maternal level covariates including the age of the mother, their monthly earnings, and education. Model 4 was finally adjusted for household-level covariates, including wealth index, food insecurity, and household size.

**Table S2:** **Sensitivity analysis showing the association between maternal body-mass index, decision-making autonomy, and adolescent nutritional status**

| **Statistical model** | **Hb status** | | **HAZ** | | **BAZ** | |
| --- | --- | --- | --- | --- | --- | --- |
|  | **β ± S.E** | **P-value** | **β ± S.E** | **P-value** | **β ± S.E** | **P-value** |
| **Model 1: crude** |  |  |  |  |  |  |
| Maternal BMI category |  |  |  |  |  |  |
| Normal (ref) |  |  |  |  |  |  |
| Underweight | 0.50 ± 0.19 | 0.01 | -0.29 ± 0.17 | 0.08 | 0.11 ± 0.13 | 0.39 |
| Overweight/obese | 0.08 ± 0.08 | 0.32 | 0.20 ± 0.06 | 0.0004 | 0.16 ± 0.04 | 0.0002 |
| Decision-making | -0.13 ± 0.03 | 0.0001 | 0.06 ± 0.03 | 0.03 | -0.05 ± 0.02 | 0.009 |
| **Model 2** |  |  |  |  |  |  |
| Maternal BMI category |  |  |  |  |  |  |
| Normal (ref) |  |  |  |  |  |  |
| Underweight | 0.00 ± 0.00 | 0.62 | 0.00 ± 0.00 | 0.64 | 0.00 ± 0.00 | 0.63 |
| Overweight/obese | 0.00 ± 0.00 | 0.63 | 0.00 ± 0.00 | 0.54 | 0.00 ± 0.00 | 0.61 |
| Decision-making | -0.00 ± 0.00 | 0.66 | -0.00 ± 0.00 | 0.59 | 0.00 ± 0.00 | 0.66 |
| **Model 3** |  |  |  |  |  |  |
| Maternal BMI category |  |  |  |  |  |  |
| Normal (ref) |  |  |  |  |  |  |
| Underweight | -0.12 ± 0.09 | 0.19 | -0.11 ± 0.09 | 0.21 | -0.11 ± 0.09 | 0.20 |
| Overweight/obese | -0.01 ± 0.05 | 0.83 | -0.02 ± 0.05 | 0.67 | -0.01 ± 0.04 | 0.85 |
| Decision-making | 0.17 ± 0.05 | 0.001 | 0.17 ± 0.05 | 0.001 | 0.16 ± 0.05 | 0.001 |
| **Model 4** |  |  |  |  |  |  |
| Maternal BMI category |  |  |  |  |  |  |
| Normal (ref) |  |  |  |  |  |  |
| Underweight | 0.01 ± 0.01 | 0.62 | 0.005 ± 0.01 | 0.65 | 0.004 ± 0.01 | 0.71 |
| Overweight/obese | 0.01 ± 0.01 | 0.46 | 0.004 ± 0.01 | 0.53 | 0.002 ± 0.006 | 0.71 |
| Decision-making | 0.001 ± 0.01 | 0.92 | 0.001 ± 0.01 | 0.92 | 0.0002 ± 0.01 | 0.97 |

Model 1 was the crude model; Model 2 was adjusted for adolescent level covariates including age, menarche, dietary diversity, and frequency of animal source intake; Model 3 was a further adjusted model for maternal level covariates including the age of the mother, their monthly earnings, and education. Model 4 was finally adjusted for household-level covariates, including wealth index, food insecurity, and household size.
